# Engineering FKBP-Based Destabilizing Domains to Build Sophisticated Protein Regulation Systems

**DOI:** 10.1371/journal.pone.0145783

**Published:** 2015-12-30

**Authors:** Wenlin An, Rachel E. Jackson, Paul Hunter, Stefanie Gögel, Michiel van Diepen, Karen Liu, Martin P. Meyer, Britta J. Eickholt

**Affiliations:** 1 MRC Centre for Developmental Neurobiology, King’s College London, London, SE1 1UL, United Kingdom; 2 Charité - Universitätsmedizin Berlin, Cluster of Excellence NeuroCure and Institute of Biochemistry, Berlin, 10117, Germany; 3 Department of Craniofacial Development, King’s College London, London, SE1 1UL, United Kingdom; University of Nebraska Medical Center, UNITED STATES

## Abstract

Targeting protein stability with small molecules has emerged as an effective tool to control protein abundance in a fast, scalable and reversible manner. The technique involves tagging a protein of interest (POI) with a destabilizing domain (DD) specifically controlled by a small molecule. The successful construction of such fusion proteins may, however, be limited by functional interference of the DD epitope with electrostatic interactions required for full biological function of proteins. Another drawback of this approach is the remaining endogenous protein. Here, we combined the Cre-LoxP system with an advanced DD and generated a protein regulation system in which the loss of an endogenous protein, in our case the tumor suppressor PTEN, can be coupled directly with a conditionally fine-tunable DD-PTEN. This new system will consolidate and extend the use of DD-technology to control protein function precisely in living cells and animal models.

## Introduction

Phosphatase and tensin homolog located on chromosome 10 (PTEN) is one of the most commonly mutated or deleted tumor suppressors in human cancer that is also causally linked to autism spectrum disorder [1,2]. Changes in PTEN expression level leads to aberrant cell cycle progression as well as to alterations in cell migration [3,4], amongst other cellular responses. However, it is still a considerable challenge to ascertain the minimum alteration in PTEN level or activity that can cause the onset of tumor formation [5] or neurological changes [6], or whether disease states can be ameliorated by reinstalling PTEN expression. Our ability to address questions of this nature are currently limited by the lack of experimental systems that allow the targeted and reversible regulation of PTEN [7]. We set out to exploit FKBP DD-technology to control PTEN protein function in a rapid, reversible and tunable manner. FKBP is a 12kDa FK506 binding protein, which is broadly expressed in various tissues and functions as a protein chaperone for newly synthesized polypeptides [8]. An engineered human FKBP has been developed that is able to bind a small synthetic ligand, Shld1, with 1000-fold selectivity over wild-type FKBP due to an amino acid (aa) substitution at F36 (F36V) [9]. A further aa substitution at L106 (L106P) causes FKBP to degrade rapidly in the absence of Shld1 [10]. Here, we further modified FKBP^F36V/L106P^ by neutralizing highly charged surface amino acid residues, thereby eliminating the ability of the DD to interfere with electrostatic interactions required for full biological function of proteins. In case of PTEN, we demonstrate that FKBP^F36V/L106P/E31S/D32S^-PTEN permits rapid, reversible and tunable regulation of PTEN function in cellular and zebrafish models. We further eliminated a potential Cre pseudo-cleavage site within the FKBP* gene sequence to render the system compatible with the Cre-LoxP system. In conjunction, the two components will facilitate the study into protein network function, as FKBP-tagged proteins and conditional mouse alleles can be combined as required by the experimental set-ups.

## Material and Methods

All animal procedures were approved by the local Animal Welfare and Ethics Review Body (King’s College London) and were carried out in accordance with the Animals (Scientific Procedures) Act 1986, under license from the United Kingdom Home Office.

### Materials and cloning

Rabbit anti-PTEN (138G6), anti-AKT and anti-pAKT (Ser473) antibodies were from Cell Signaling Technology; the mouse anti-PTEN (A2B1) from Santa Cruz Biotechnology; mouse monoclonal anti GAPDH from Abcam. Phalloidin-Alexa fluorophores conjugates and anti-rabbit secondary antibody were obtained from Molecular Probes. pCDNA3+ (Invitrogen) and pCAG vector (a gift from Dr. Kobayashi Mime) were modified by adding *Hin*dIII and *NotI* sites. The FKBP*-PTEN cassettes for linker optimization or surface engineering were cloned into pcDNA3+ or pCAG by *HindIII* and *NotI* sites. The construct pCAG-FKPTEN was generated by cloning a Neomycin resistance gene cassette and the FKBP**-PTEN open reading frame into the pCAG vector. The CTV vector (Addgene) was used to create constructs M3L^cta^ and M3L^ttg^ by inserting FKBP**-PTEN gene at the *AscI* site.

### Cell culture

PTEN null U87MG cells were cultured in Dulbecco’s Modified Eagle Medium (DMEM) media containing 10% FCS and 1% penicillin/streptomycin. The U87MG cell line stably expressing FKBP**-PTEN was generated by G418 selection (500 μg/ml) and maintained in DMEM media containing 10% FCS and G418 (250 μg/ml). Cells were maintained at 37°C, 5% CO_2_ in a humid atmosphere. Cerebral cortices were dissected from either CD1 or PTEN^flox/flox^ E14.5 mouse embryos, dissociated in 0.33 mg/ml trypsin (Worthington, UK) in HBSS (Gibco, UK) for 15 min at 37°C and gently triturated with a fire polished Pasteur pipette in Neurobasal medium (Gibco, UK) supplemented with 2% fetal calf serum (FCS), 2% B27, 1% Glutamax and 1% penicillin/streptomycin (P/S). Neurons seeded at a density of 2000 cell/mm^2^ on poly-L-lysine coated culture plates.

### Immuofluoresence staining

U87MG cells stably expressing FKBP**-PTEN were cultured on coverslips and fixed in 4% PFA in PBS for 20 min. Cells were then washed with PBS and blocked in blocking buffer (0.5% Triton X-100, 2% bovine serum albumin and 1% goat serum in PBS) for 1 h. Cells were incubated with rabbit anti-PTEN for 1h at room temperature and washed 3 times in PBS, and incubated with an Alexa Fluor^®^ 488 anti-Rabbit IgG, Alexa Fluor^®^ 568 Phalloidin and Hoechst for 1 h at room temperature. Cells were then washed three times in PBS and mounted using Mowiol^®^ mounting medium.

### Transfection and nucleofection

**Fig 1:**
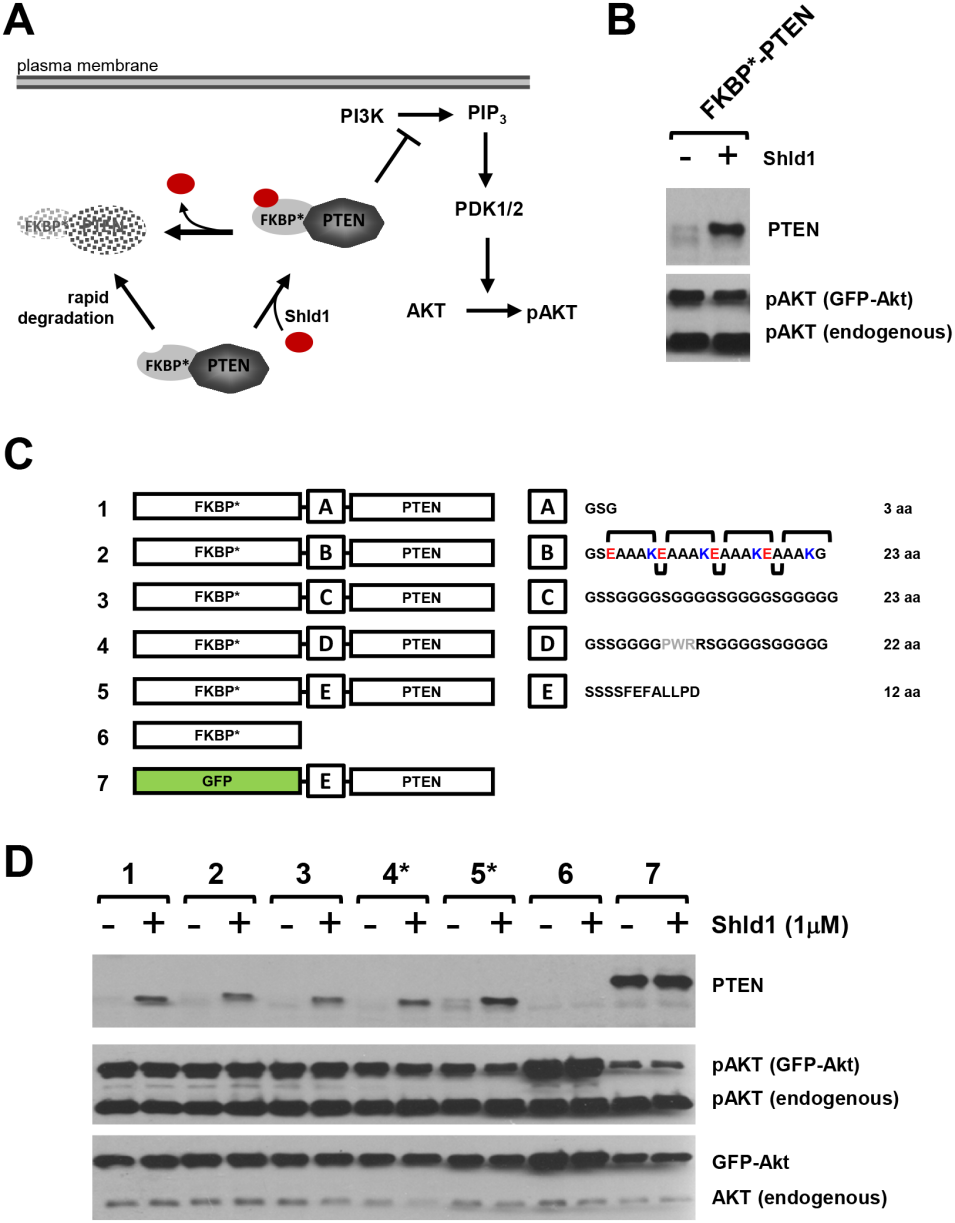
Consequence of linker optimization on PTEN activity of the FKBP*-PTEN fusion protein. (**A)** Rationale of the DD-technology: Fusion of PTEN with a modified human FKBP variant harboring the F36V and L106P point mutations (FKBP*) leads to degradation of the fusion protein. Presence of the FKBP* ligand Shld1 confers stabilisation of FKBP*-PTEN and results in inhibition of PI3K/Akt signaling. (**B)** The fusion protein FKBP*-PTEN shows no PTEN activity. PTEN-deficient U87MG cells were transiently co-transfected with FKBP*-PTEN and AKT-GFP. After one day, cells were treated with Shld1 for 12 hours at 1μM before cell lysis and samples analyses by western blotting using indicated antibodies. **(C)** Consequence of linker optimization on PTEN activity of the FKBP*-PTEN fusion protein. Schematic representation of linker optimization. FKBP*-linker-PTEN variants with different linker regions (A-E) were generated. Linker A—short flexible linker; Linker B—a helix forming linker. Linker C—long flexible linker. Linker D—long flexible linker containing the PWR motif. Linker E—the original linker present in the enzymatic active GFP-PTEN. **(D)** Analyses of the effect of different linkers on PTEN activity in FKBP*-PTEN fusion variants in U87MG cells. Constructs expressing FKBP*-PTEN variants or GFP-PTEN were co-transfected with AKT-GFP into a PTEN null U87MG cells. Stabilization of FKBP*-PTEN or GFP-PTEN in presence or absence of Shld1 was monitored by PTEN antibody. The phosphorylation level of AKT-GFP at Ser473 served as a fast and effective experimental protocol to control for PTEN activity towards PIP3-dependent signaling in transfected cells, only. Only constructs 4 and 5, which contain proline in the linker sequence, showed moderate PTEN activity towards PI3K signaling.

**Fig 2:**
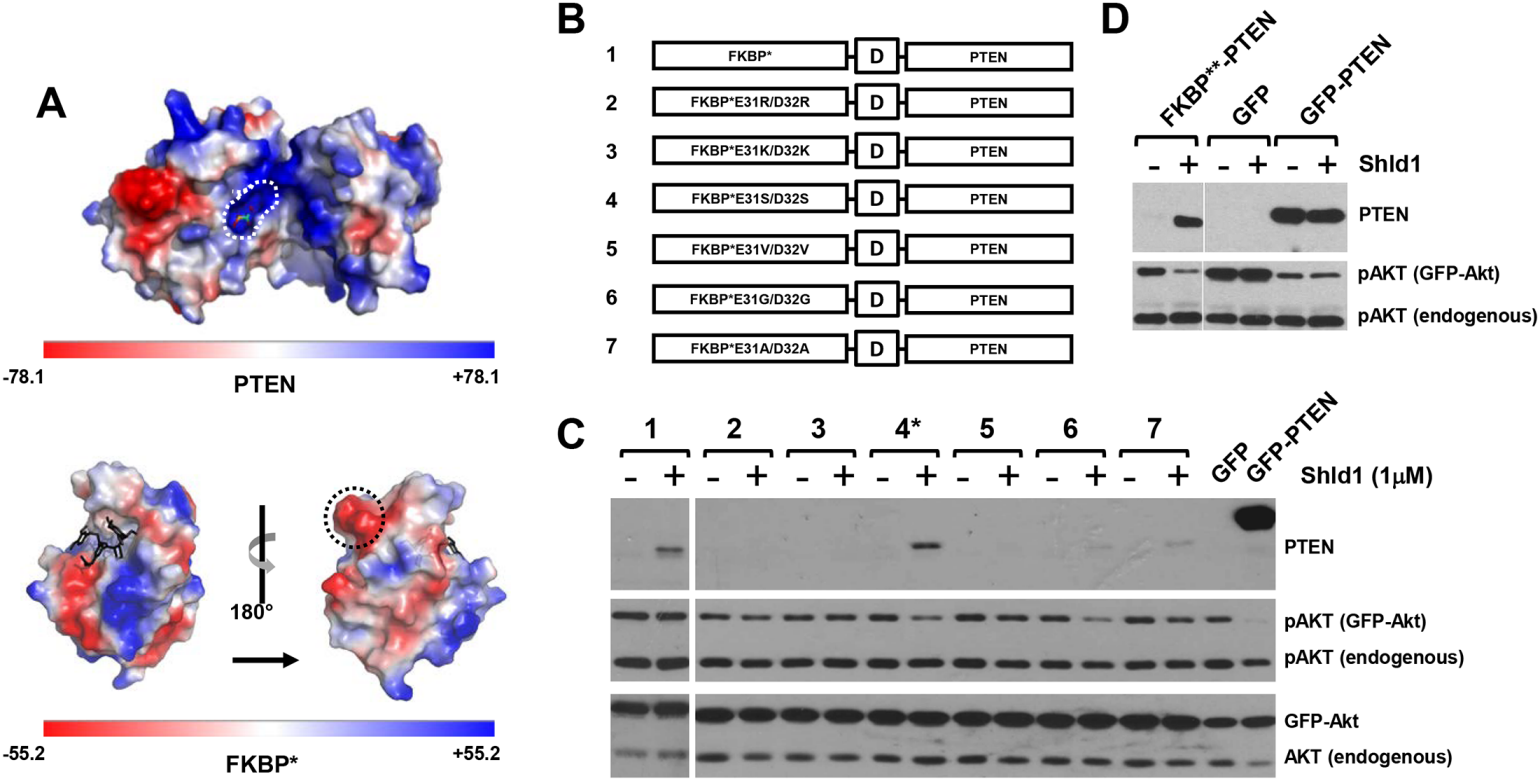
FKBP surface engineering restores PTEN activity of FKBP**-PTEN fusion protein. **(A)** Electrostatic potential map of PTEN (PDB ID: 1d5r) and FKBP (PDB ID: 1BL4). Positively charged binding pocket of the PTEN PIP3 substrate is circled with a white dashed line; the negatively charged lump formed by E31/D32 residues on the FKBP surface is circled by a black dashed line. **(B)** Schematic representation of amino acid residue substitutions of FKBP* protein surface at E31/D32. **(C)** Different FKBP*-PTEN mutants were transiently co-tansfected with AKT-GFP into U87MG cells, the expression of fusion protein and pAKT were examined by Western blotting. **(D)** E31S/D32S amino acid substitutions on FKBP* (FKBP**) restores PTEN activity of the FKBP**-PTEN fusion protein. FKBP**-PTEN (or GFP/GFP-PTEN) was transiently co-expressed with AKT-GFP in U87MG cells. Cells were treated with Shld1 and analyzed as before.

**Fig 3:**
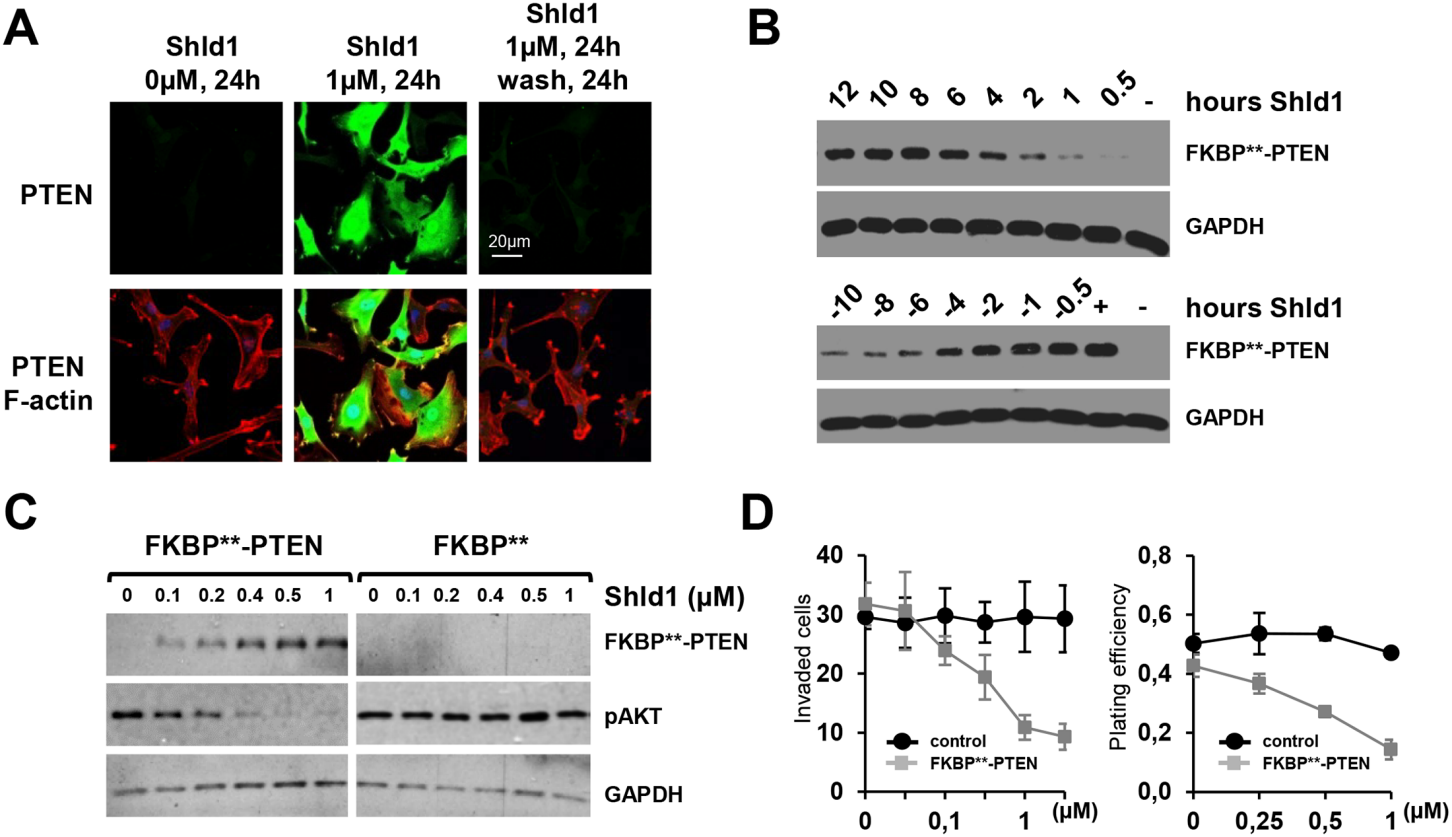
Engineered FKBP** permits rapid, reversible and tunable regulation of PTEN function in cellular models. **(A)** FKBP**-PTEN is reversibly regulated in response to Shld1 treatment in cells. U87MG cells stably expressing FKBP**-PTEN were treated with vehicle or with Shld1 (1μM) for 24 h. One set of cells was then washed thoroughly with DMEM and cultured in Shld1-free culture media for further 24hs. The expression level of FKBP**-PTEN was examined by immunofluorescence staining. **(B)** upper panel, U87MG cells stably expressing FKBP**-PTEN were treated with Shld1 (1 μM) for different time periods; lower panel, Shld1 (1 μM) was removed after 24 h treatment, and cultured in Shld1 free culture media for different time periods as indicated. **(C)** PTEN protein levels and activity can be maintained at specific concentrations over a prolonged period of time in dependence of Shld1. U87MG cells stably expressing FKBP**-PTEN were cultured in media containing indicated concentrations of Shld1 for 24 h. **(D)** Stable FKBP**-PTEN expressing U87MG cells were analyzed in cell invasion (left) and colony formation (right) assays. In the cell invasion assay, data are shown as the mean ±SEM of the number of cells migrated in three independent experiments. Plating efficiency refers to the ratio of the number of colonies to the number of cells seeded, shown as mean ±SEM of three independent experiments.

Transfection of plasmid DNA into U87MG cells was carried out using GeneJuice (Novagen). Nucleofection of mouse primary cortical neurons was performed by using Amaxa^*®*^ Mouse Neuron Nucleofector^*®*^ Kit *(*Lonza, UK). In order to monitor PTEN activity in transiently transfected U87MG cells, the co-transfection of AKT-GFP with FKBP*-PTEN or GFP-PTEN constructs was exploited as a fast and effective experimental protocol that allowed the analysis of PTEN activity towards PIP3-dependent signaling in transfected cell, only. This strategy was pursued in transient transfections (Figs 1 and 2), but not in stable FKBP**-PTEN expressing U87MG cells lines (Fig 3).

### Western blotting

U87MG cells or primary neuron cultures was washed with ice-cold PBS and then lysed in lyses buffer (50 mM Tris-HCl, pH7.5, 150 mM NaCl; 1% Triton X-100; 500 μM NaF; 1 mM sodium orthovanadate; 1X protease inhibitor) for 10 min. Cell debris was removed by centrifugation at 13000 rpm for 10 min. The boiled supernatants supplemented with SDS loading sample buffer were loaded and run on 8% SDS-PAGE gel. Separated proteins were transferred to a nitrocellulose membrane. Blots were blocked in 5% skimmed milk powder in TBST (20 mM Tris; 150 mM NaCl; 0.1% Tween) for 1 h and incubated with primary antibody in 5% skimmed milk powder/TBST overnight at 4°C or for 1h at room temperature. The expression of proteins was visualized by HRP-conjugated secondary antibody and enhanced Chemiluminescence Kit (Pierce).

### Colony formation assay

A single-cell suspension was prepared in 0.35% low melting point agar and plated (100 or 200 cells per well) in 24 well plates, which were pre-coated with 0.5% agar. Cell were then cultured for 3 weeks in media containing different concentrations of Shld1. Experiments were analyzed according to the protocol published by Franken *et al*. [11]. Plating efficiency was determined by the ratio of the number of colonies to the number of cells seeded.

### Cell invasion assay

Cell invasion assay was performed using BioCoat^™^ invasion chamber (BD Biosciences) with 8μm pore, according to the manufacturer’s instructions (BD Biosciences) with minor modifications. 0.5 ml of cell suspension (2.5x10^4^ cells per well) prepared in serum-free media was added on rehydrated 24-well BioCoat^™^ Matrigel^™^ Invasion Chambers, which were placed in 24-well plate containing normal culture media (DMEM plus 10% FBS), and incubated in a humidified tissue culture incubator for 19 h, at 37°C, 5% CO_2_ atmosphere. The cells were fixed with 4% PFA, permeabilized with 100% methanol for 20 min and stained with 0.05% crystal violet for 10 min. The cells and matrigel on the upper surface of the Matrigel Invasion Chambers were removed by scrubbing with cotton buds. Invasive cells were counted under microscope in 10 fields for each treatment.

### Zebrafish microinjection and visualization of FKBP**-PTEN expression

U87MG cells stably expressing FKBP**-PTEN were transiently transfected with RFP or pre-stained with DiI (Sigma). Approximately 100 cells were then microinjected into the perivitelline cavity of 2 dpf (day of post fertilization) zebrafish larvae. Fish larvae were immediately imaged under an inverted fluorescence microscope (0 dpi) and then transferred into Danieau’s solution containing 4 μM Shld1 or control media containing no Shld1. Fish larvae were again imaged after 24h (1dpi) to analyze the effect of Shld1 induced FKBP**-PTEN stabilization on tumor growth and migration.

FKBP**-PTEN stabilization in fish larvae was verified by immuostaining with PTEN antibody. Briefly, embryos were fixed in 4% PFA in PBS overnight at 4°C, and then sequentially dehydrated with 15% and 30% sucrose. Cryosections were stained by immunofluorescence staining protocols. Images were obtained by confocal microscopy.

### Statistical analysis

The migration of the injected U87MG cells stably expressing FKBP**-PTEN in fish embryos in presence or absence of Shld1 were analysed in a similar way to fluorescent protein diffusion profiles [12] using the Radial Profile Plot plug-in of ImageJ. The concentric movement of cells away from a central point was quantified as a measure of increased fluorescence intensity at a distance from the central point over time. Curve fitting was carried out in Excel using the equation: *f(t)* = *b*_*0*_ exp_(-b1)(t)_ where *b*_*0*_ and *b*_*1*_ are parameters of the curve. Migration index (Mi) was calculated by as a ratio of *b*_*0*_ at time points 1dpi and 0dpi (*Mi* = *b*_*0(1dpi)*_ */b*_*0(0dpi)*_). The average migration index of control group (without Shld1 treatment, n = 13) is compared with that of Shld1 treated group (n = 11). The size of tumor mass was measured by ImageJ. The average ratio of tumour mass size (1 dpi/0 dpi) was calculated and analyzed by Excel® and SPSS using student two tailed *t*-*test* **p<0.01; *** p<0.001.

## Results

We fused PTEN at the C-terminus of the FKBP^F36V/L106P^-based DD (FKBP^F36V/L106P^ will be referred to as FKBP*) using a 12 aa linker sequence (-SSSSFEFALLPD-). We proposed that in the absence of Shld1, FKBP* would be rapidly degraded by the ubiquitin proteasome system [13], resulting in the loss of FKBP*-PTEN fusion protein, whereas addition of Shld1 will stabilize the protein and inhibit PI3K/Akt signaling (Fig 1a). Although expression levels of the FKBP*-linker-PTEN fusion demonstrated Shld1 dependency, the protein did not alter PI3K/Akt signaling (Fig 1b). FKBP* has been successfully used to regulate a number of different proteins, including cdc42 [14], RhoA [14], and MLYCD [15], with no reported interference in their activity. We therefore surmised that the linker might not be suitable for generating biological active FKBP*-PTEN. Based on available knowledge regarding linker optimization [16,17], we composed a number of different peptide sequences with the aim of restoring PTEN activity in the FKBP*-linker-PTEN fusion protein (Fig 1c). However, the most theoretically effective linkers [17], a helix forming linker or a long flexible linker, did not restore PTEN activity as verified by analyzing pAkt levels in PTEN-null U87MG glioblastoma cells transfected with linker variants (Fig 1d). In contrast, a linker containing the unique cyclic imino acid proline, which is unable to form hydrogen bonds to surrounding amino acids, was able to slightly improve PTEN activity (linker sequence 4, Fig 1d). This limited success in restoring PTEN activity using linker optimization suggest that other mechanisms impede biological function of the phosphatase when presented in the FKBP*-PTEN fusion protein.

### FKBP surface engineering restores PTEN activity of the FKBP-PTEN fusion protein

We considered the possibility that FKBP* might directly interfere with PTEN activity by blocking substrate access to the active site of the enzyme, which consists of a large pocket with positive charges that accommodates the PTEN phosphoinositide substrates (Fig 2a). Indeed, the electrostatic potential maps of the PTEN surface (PDB ID: 1d5r) and the FKBP* surface (PDB ID: 1BL4) revealed complementary charge distributions of the PTEN substrate binding region and large negatively charged protrusions present on the FKBP* surface (Fig 2a). Therefore, we engineered the FKBP* surface such that the charged regions were neutralized by replacing two acidic amino acid residues (E31 and D32) with basic amino acid residues (Arg or Lys), with polar uncharged residues (Ser), with hydrophobic residues (Val or Ala), or with the small amino acid Gly (Fig 2b). Transfection of FKBP*-PTEN surface engineered variants into U87MG cells demonstrated that FKBP* modifications containing the aa substitutions E31S/D32S, E31G/D32G or E31A/D32A (Fig 2b, PTEN variants 4, 6, 7) resulted in Shld1 dependent regulation of PTEN activity towards PI3K/Akt signaling. However, only the E31S/D32S FKBP* mutant was efficiently stabilized by Shld1 (Fig 2c). Hereafter, we refer to FKBP*^E31S/D32S^-PTEN as FKBP**-PTEN. As the expression level of stabilized FKBP**-PTEN defines the dynamic range of the scalability of the FKBP**-PTEN regulation system, we exploited the strong CAG promoter sequence, a combination of the CMV early enhancer element and the chicken beta-actin promoter to drive FKBP**-PTEN expression. Subcloning of the FKBP**-PTEN cassette into a pCAG vector increased the level of protein expression and activity of FKBP**-PTEN to a level comparable to GFP-PTEN (Fig 2d).

### Engineered FKBP** permits rapid, reversible and tunable regulation of PTEN function in cellular and zebrafish models

To further characterize the scalability, speed and reversibility of FKBP**-PTEN in response to Shld1, we generated a U87MG cell line stably expressing FKBP**-PTEN under the control of the CAG promoter (Fig 3). Immunofluorescence labeling of these cells revealed that FKBP**-PTEN was robustly stabilized following the addition of Shld1, and this stabilization could be abolished by Shld1 washout (Fig 3a and 3b). FKBP**-PTEN protein levels were gradually up-regulated with increasing Shld1 concentration, resulting in a concomitant decrease in phosphorylated Akt, indicating that PI3K signaling can be fine-tuned by altering the level of FKBP**-PTEN (Fig 3c). The time-dependent stabilization profile of FKBP**-PTEN showed that the engineered FKBP** responded within a few hours of addition of Shld1 and reached a plateau after 8 h (Fig 3b). Thus, the stabilization kinetics of FKBP** is comparable to, or even faster than that of FKBP* [14]. We also discovered a rapid turnover of FKBP**-PTEN within a few hours after the removal of Shld1 (Fig 3b). The half-life of FKBP**-PTEN fusion protein is as short as 2-4h, which is comparable to FKBP* [15] and to approximately 5% of the 48–72 h half-life of endogenous PTEN [18]. Thus, our engineered FKBP** provides an efficient molecular tool to manipulate PTEN activity in a scalable, rapid and reversible manner. To characterize whether our engineered FKBP** could finely tune PTEN activity and regulate biological responses, we carried out *in vitro* invasion and colony formation assays [11,19] using the FKBP**-PTEN U87MG cell line. Cell invasion and colony formation in FKBP**-PTEN U87MG cells, but not wild type U87MG cells, could be inhibited by Shld1 in a dose-dependent manner (Fig 3d). Thus, Shld1 can be used to fine tune PTEN activity to inhibit tumor growth and tumor cell division.

**Fig 4:**
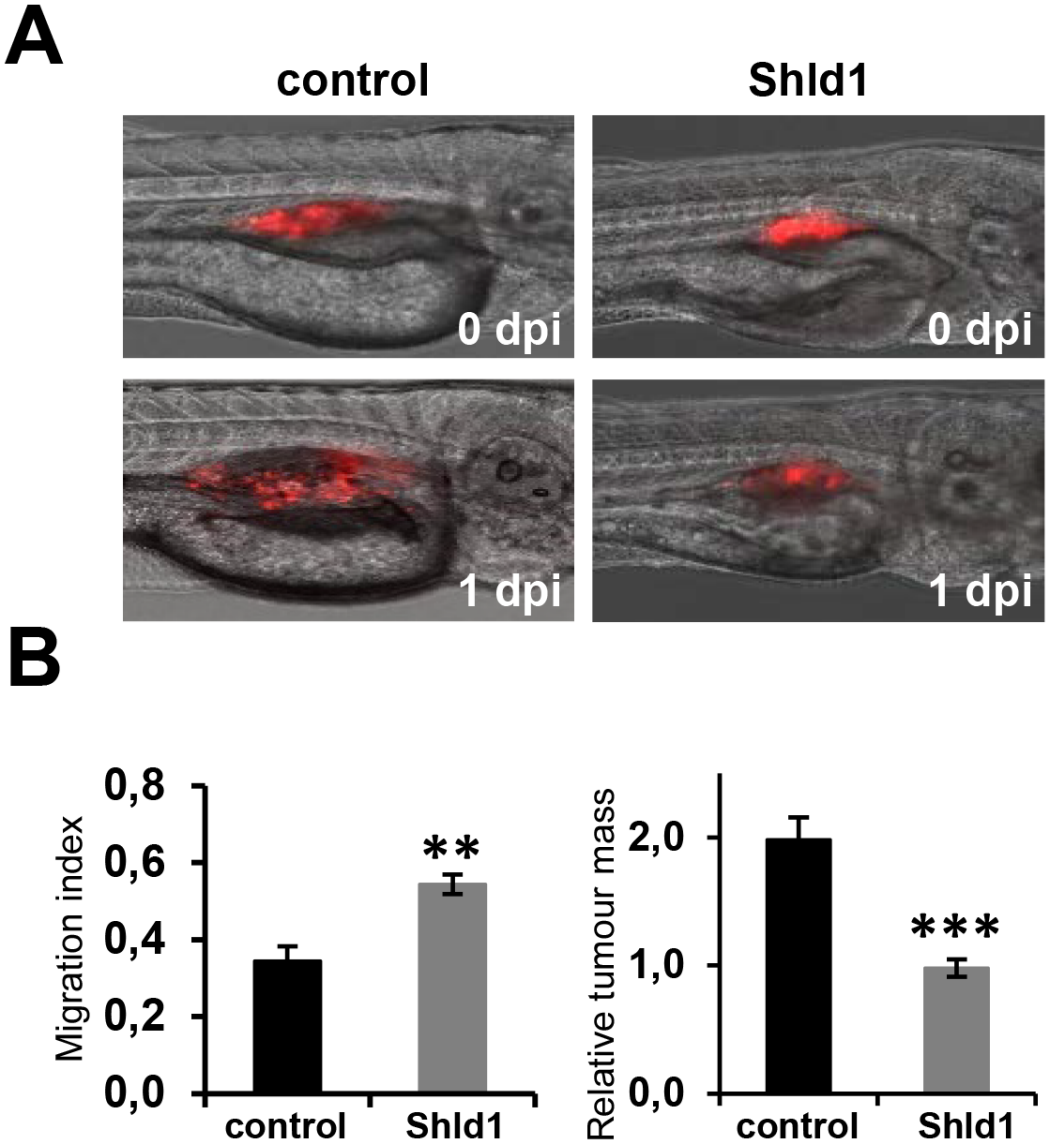
Engineered FKBP** permits regulation of PTEN function in the zebrafish. **(A)** The migration and growth of stable FKBP**-PTEN expressing U87MG cell clones injected into zebrafish embryos can be inhibited by Shld1. Prior to microinjection into the perivitelline cavity of 2 days post fertilization (dpf) zebrafish embryos, cells were pre-stained with DiI. Embryos were then transferred into fish water with (n = 11) or without (n = 13) Shld1 (4 μM) and maintained for 24h. Images were taken immediately after injections (0 days post injections, dpi) and after 24 h (1dpi). **(B)** The migration indices and the increase in tumor mass size of transplanted cells were quantified. ** p<0.01; ***p<0.001.

To investigate whether Shld1 can induce FKBP**-PTEN stabilization and control biological responses *in vivo*, we used an established zebrafish metastasis model [20], and microinjected the FKBP**-PTEN U87MG cells, either pre-stained with DiI or transfected with RFP, into the perivitelline cavity of 2 days post fertilization (dpf) zebrafish larvae. Larvae were then transferred into Danieau’s solution with or without 4μM Shld1. Images of larvae were taken immediately after injections and 24h post-injection (Fig 4a). The migration indices and the increase in tumor mass size of transplanted cells were quantified over this time-course. These experiments demonstrated that Shld1 is effective in stabilizing FKBP**-PTEN in zebrafish larvae, resulting in inhibition of invasion and growth of FKBP**-PTEN U87MG cells (Fig 4b). They also show for the first time that DD-systems can be used effectively to manipulate protein levels on demand in the zebrafish.

### Codon optimization of FKBP** establishes a conditional fine-tuning protein regulation system

**Fig 5:**
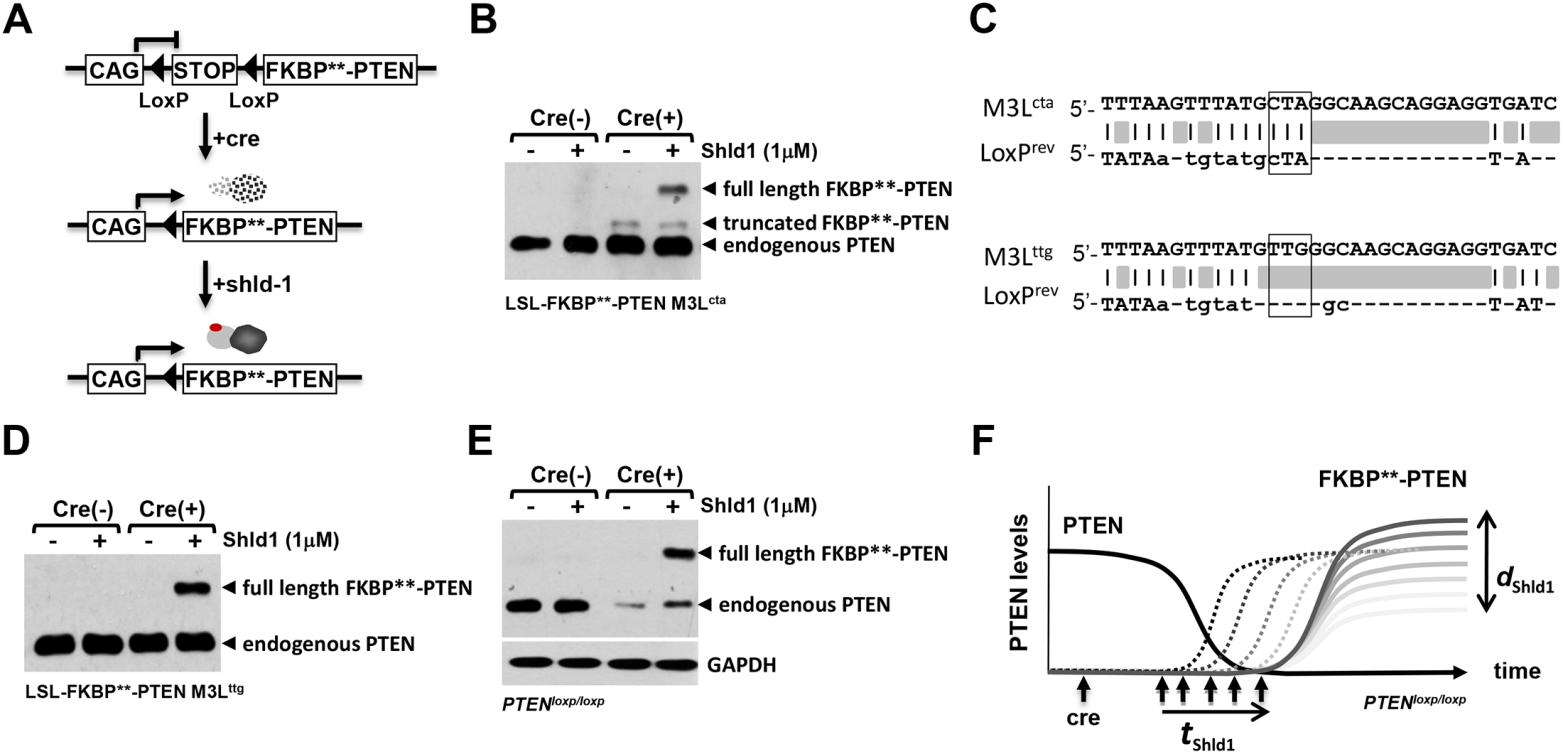
Codon optimization of FKBP** establishes a conditional fine-tuning protein regulation system. **(A)** The transcription of the destabilization cassette FKBP**-PTEN was subcloned downstream of LoxP-Stop-LoxP; transcription was driven by the ubiquitous CMV-enhanced chicken beta actin promoter (CAG). Presence of Cre induces FKBP**-PTEN gene transcription; however, the translated FKBP**-PTEN will be rapidly degraded. Addition of Shld1 confers FKBP**-PTEN stabilization in a tunable and reversible manner. **(B)** Cre-mediated cleavage of LoxP-Stop-LoxP (LSL) created a truncated FKBP**-PTEN fusion protein. Mouse forebrain neurons were nucleofected with LSL-FKBP**-PTEN and Cre-IresRFP (Cre (+)), or RFP (Cre (-)). Cells were treated with Shld1 (or control vehicle) overnight, before analyses of cell lysates using indicated antibodies. **(C)** Sequence alignment of FKBP** and LoxP. The third ATG codon region sequence (TATGCTA) of FKBP** (M3L^cta^) is identical to the linker region of the LoxP stem-loop sequence (TATGCTA). Codon optimization of CTA with TTG (both code for amino acid Leu) will destroy the potential pseudo-cleavage site of Cre on FKBP**. **(D)** Codon optimization of M3L^cta^ to M3L^ttg^ in FKBP** abolished the Cre-dependent FKBP**-PTEN truncation and produced a Shld1 dependent PTEN fusion protein. The M3L^ttg^ modified LSL-FKBP**-PTEN construct was co-expressed with Cre-IresRFP (Cre (+)), or RFP (Cre (-)) in mouse forebrain neurons as before. **(E)** Combinatorial use of the Cre-LoxP system with the FKBP**-PTEN/Shld1 chemical-genetic protein control system in PTEN^flox/flox^ cells. Constructs were nucleofected into *PTEN* ^*loxp/loxp*^ mouse primary neurons as before. Note that nucleofection of primary cells occurs with efficiencies at approx. 80%, and result in a residual endogenous PTEN signals detected by western blotting. **(F)** The generated system is able to couple PTEN-loss directly with the expression of tunable FKBP**-PTEN. Upon Cre-mediated recombination, *PTEN* ^*loxp/loxp*^ cells will lose PTEN and, at the same time, activate expression of FKBP**-PTEN. In essence, endogenous PTEN is replaced by tuneable PTEN, which will enable to test whether PTEN-loss induced phenotypes can be rescued at different time-points (*t*_Shld1_) and/or at different PTEN-concentrations (*d*_Shld1_).

In order to achieve advanced regulation of protein function, it would be desirable to combine tunable FKBP**-PTEN with established systems enabling precise control of gene expression, such as the Cre/LoxP system (Fig 5a). For this purpose, FKBP**-PTEN was subcloned immediately downstream of the second LoxP site of the LoxP-Stop-LoxP (LSL) cassette. We tested the pCAG-LSL-FKBP**-PTEN construct in mouse cortical neuronal primary cultures by transient co-transfection with Cre-IresRFP (or, in control experiments, RFP). The FKBP**-PTEN fusion protein was only detectable by western blotting in the presence of Cre, and following application of the synthetic ligand Shld1 (Fig 5b). Surprisingly, when Cre and FKBP**-PTEN were co-expressed, the anti-PTEN antibody also identified a distinct protein band with lower molecular weight than full-length FKBP**-PTEN, which was stable even in the absence of Shld1. We reasoned that this protein could represent a form of FKBP**-PTEN with a truncation in the FKBP** domain which allows it to escape Shld1 regulation. A mechanism involving Cre-dependent truncation of FKBP**-PTEN suggests that these two regulation systems could be incompatible. Due to the sequence similarity of FKBP* and the engineered FKBP** domain it is likely that this might also be true of the parental DD system. We therefore investigated the potential sites of Cre action within the DD domain.

Cre recombinase is an enzyme known to catalyze site specific recombination events between two DNA recognition sites (LoxP sites) of 34 base pairs, which form a stem-loop-stem structure. We aligned the LoxP sequence with the human FKBP** DNA sequence and found an 88.9% similarity between the loop region of LoxP and the sequence surrounding the third ATG of the human FKBP** gene (M3L^cta^ and LoxP^rev^) (Fig 5c). Consequentially, the third ATG region (ATGCTA) of the human FKBP** gene might be erroneously recognized as a Cre cleavage site, resulting in the formation of a FKBP gene truncation in which translation begins from the fourth ATG. In this case, although the destabilizing site L106P on FKBP** protein still remains, the truncated FKBP** might lose its destabilizing property as shown by the Shld1-independent band (Fig 5b). To test this idea, we replaced the high similarity region ATGCTA (M3L^cta^) of the human FKBP** gene with silent mutations ATGTTG (M3L^ttg^). We found that the truncated FKBP**-PTEN band (Fig 5b) disappeared following mutations of L^cta^ to L^ttg^, whilst the expression of full-length FKBP**-PTEN remained both Cre- and Shld1-dependent (Fig 5d). Thus, we engineered FKBP** further to implement a functioning system that offers dual control of FKBP**-PTEN expression at transcription and posttranslational levels.

In order to test if PTEN loss can be directly coupled with the expression of tunable FKBP**-PTEN, we co-transfected M3L^ttg^ pCAG-LSL-FKBP**-PTEN with Cre-RFP (or RFP alone in control experiments) into PTEN^flox/flox^ mouse neurons by nucleofection. Cre expression led to substantial decreases in endogenous PTEN levels, and created Shld1 depended regulation of exogenous FKBP**-PTEN (Fig 5e). Thus, insertion of the LSL cassette ensures that loss of endogenous PTEN and gain of tunable FKBP**-PTEN takes place in the same cells.

## Discussion

The targeting of protein stability with small molecules has emerged as a strategy in synthetic biology to ascertain protein function. The technique involves genetically endowing the protein of interest with a protein destabilizing domain specifically and reversibly controlled by a small molecule, so that the stability of the fusion protein is dependent upon the sequence of the DDs rather than that of the POI. An established DD sequence is based on a modified FKBP (FKBP^F36V/L106P^); however, one of the recurring problems associated is the possible interference of the FKBP^F36V/L106P^ tag with electrostatic interactions, as well as the remaining unaffected endogenous protein, which interfered here with the phosphatase activity of PTEN.

We modified FKBP^F36V/L106P^ by neutralizing highly charged surface amino acid residues. This modification resolved the problem of electrostatic interference by ensuring the full biological activity of PTEN, which retains highly charged amino acids within the active site essential for function. We demonstrate that our newly generated fusion protein (FKBP**-PTEN) permits rapid, reversible and tunable regulation of protein function in cellular as well as, for the first time, in zebrafish models. Furthermore, we have rendered the system compatible with the Cre-LoxP system. We identified and eliminated a potential Cre pseudo-cleavage site within the FKBP gene. Following Cre-mediated recombination, expression of the FKBP**-PTEN gene is achieved. The system now provides two layers of control over protein levels and activity: the first operating at transcriptional and the second at posttranslational level.

The development of the FKBP-Shield1/Cre-LoxP system offers unique possibilities to manipulate protein function *in vitro* and *in vivo*, by combining the loss of endogenous proteins directly with the expression of tunable versions. This system is compatible with the building of sophisticated protein regulation networks as FKBP**-tagged proteins, as well as conditional mouse alleles can be selected and combined as required. Additionally, the system allows precise and effortless control over the re-installment of protein function following molecular deficits, thus offering a powerful means with which to address the reversibility of a number disease states, in particular those associated with developmental disorders. Therefore, this study consolidates and extends the use of DD-technology to manipulate protein function in a specific, fast, reversible and tunable manner.
